# Plantation Mapping in Southeast Asia

**DOI:** 10.3389/fdata.2019.00046

**Published:** 2019-12-06

**Authors:** Xiaowei Jia, Ankush Khandelwal, Kimberly Carlson, James S. Gerber, Paul C. West, Vipin Kumar

**Affiliations:** ^1^Department of Computer Science and Engineering, University of Minnesota – Twin Cities, Minneapolis, MN, United States; ^2^Department of Natural Resources and Environmental Management, University of Hawai'i Manoa, Honolulu, HI, United States; ^3^Institute on the Environment, University of Minnesota – Twin Cities, St. Paul, MN, United States

**Keywords:** remote sensing, plantation, deep learning, deforestation, ensemble learning

## Abstract

Plantation mapping is important for understanding deforestation and climate change. While most existing plantation products are created manually, in this paper we study an ensemble learning based framework for automatically mapping plantations in southern Kalimantan on a yearly scale using remote sensing data. We study the effectiveness of several components in this framework, including class aggregation, data sampling, learning model selection and post-processing, by comparing with multiple baselines. In addition, we analyze the quality of our plantation mapping product by visual examination of high resolution images. We also compare our method to existing manually labeled plantation datasets and show that our method can achieve a better balance of precision (i.e., user's accuracy) and recall (i.e., producer's accuracy).

## 1. Introduction

In recent years, biofuels synthesized from crops have provided an opportunity to reduce the dependence on fossil fuels. Biodiesel, for example, is produced using organic fats and vegetable oils, such as palm oil and can be used with petrol diesel. Biofuels may help strengthen the energy security in countries that do not have direct availability of fossil fuel deposits while reducing of greenhouse gas (GHG) emissions (Sorda et al., 2010). However, the production of biofuel crops can also have a negative impact on the environment, such as deforestation (Fargione et al., 2008). Moreover, biofuels also put more stress on water and land resources that could otherwise be used for the production of food (Cai et al., 2010). Therefore, the competing needs for land and water resources by food and biofuel production has been a very important issue of the food-water-energy debate (Tilman et al., 2009; Lambin and Meyfroidt, 2011).

A prime example of a very strong interplay between food, water and energy is the unsustainable palm oil production in Indonesia and other countries for biofuels and human consumption (Mukherjee and Sovacool, 2014). Global palm oil production is dominated by Indonesia and Malaysia, which together account for over 90% of total global palm oil (WorldAtlas, 2018). Indonesia is currently the largest producer and exporter of palm oil worldwide. Furthermore, due to the increase global population, demand for palm oil globally shows an increasing trend. This rapid growth of the palm oil industry has happened at the expense of severe damage to tropical forests which play a very critical role in the carbon cycle of the earth. Tropical forests cover only 7–10% of the Earth's surface (Malhi and Grace, 2000; Nightingale et al., 2004), but they are globally important, containing 40–50% of all carbon stored in terrestrial vegetation. The role of these forests in the global carbon cycle is important because it is estimated that tropical deforestation is responsible for 20% of global anthropogenic carbon emissions (Parry et al., 2007). Tropical rainforests on Southeast Asia are unique as they have exceptionally high biodiversity and large extent of tropical peatlands. Tropical peatlands are very rich sinks of carbon containing globally a peat carbon pool of 88.6 Gt (equal to 15–19% of the global peat carbon pool), of which 57.4 Gt carbon is in Indonesian peatlands alone (Page et al., 2011). Forest clearance, peatland drainage and fires (both natural and caused by humans) lead to significant emissions of CO_2_ to the atmosphere. For example, 1997–98 Indonesian peatland fire released more than 0.87 Gt of carbon, equivalent to 14% of the average annual global fossil fuel emissions released during the 1990s (Page et al., 2002).

Apart from biodiversity loss and high carbon emissions, the large scale at which palm oil is cultivated can also lead to poor soil and water quality and availability problems. For example, palm oil mill effluent (POME) has been disposed of as untreated waste into natural water sources and has caused severe degradation of water quality. Furthermore, the general oil extraction process is water intensive, as it demands large quantities of water (Sheil et al., 2009; Rupani et al., 2010).

The unsustainable nature of plantation industry in Southeast Asia and other parts of the world has been acknowledged globally. Various companies and governments are trying to ensure that the palm oil plantations meet rigorous sustainability standards (Scarlat and Dallemand, 2011). For example, under a new European Union biofuel policy, any palm oil biodiesel imported to the region must, over its full life cycle, demonstrate a 35% savings in greenhouse gas emissions compared to fossil fuel diesel, and the feedstock cannot be grown in areas with high biodiversity value or a high stock of carbon. Major corporate groups in Indonesia and other Southeast Asian countries have also started moving toward sustainable production of palm oil through certification under the Roundtable on Sustainable Palm Oil (RSPO) (Schouten and Glasbergen, 2011) and other certification programs, such as International Sustainability and Carbon Certification (ISCC) (Moser et al., 2014) and ISPO certification (Paoli et al., 2013). However, evaluating the effectiveness of these diverse policies depends on the ability to monitor land cover change due to plantation expansion (Carlson et al., 2018). Hence, scalable and timely monitoring of these land uses is essential for understanding whether programs and policies are meeting their stated goals (WCS, 2010; Wakker and Asia, 2014).

Classification of remotely sensed images into different land cover classes has been a widely used approach in various earth science applications. Remote sensing data from various earth observation satellites, such as the Advanced Very High Resolution Radiometer (NOAA AVHRR), Satellite Pour l'Observation de la Terre (SPOT) VEGETATION, Moderate Resolution Imaging Spectroradiometer (MODIS), and LANDSAT, have been used to produce forest and land cover maps for large-scale land cover monitoring (Achard and Estreguil, 1995; Mayaux et al., 1998; Hansen et al., 2000).

In particular, remote sensing data acquired through various earth observation satellites provide immense opportunity to monitor land use/land cover (LULC) changes caused by plantation cultivation. However, current state of the art methods based on remote sensing data are limited in their temporal frequency and scalability due to various reasons, such as need for human intervention, use of very simple machine learning methods and are applicable only for small regions (Hansen et al., 2008, 2013; Hoscilo et al., 2011; Dong et al., 2012; Li and Fox, 2012; Margono et al., 2012; Miettinen et al., 2012a,b; Ziegler et al., 2012; Gutiérrez-Vélez and DeFries, 2013). To this date, there is no existing framework that can provide plantation extent maps in an automated fashion at yearly scales for large regions.

Even though yearly plantation maps are not available, a few organizations have developed plantation maps for a single or a few years. Two such datasets are Tree Plantation (TP) dataset (Petersen et al., 2016) and RSPO dataset (Gunarso et al., 2013). These datasets have reasonable accuracy as they have been prepared using visual interpretation by human experts. The TP dataset provides the location of tree plantations in selected tropical countries circa 2013–2014. According to the visual inspection conducted by Petersen et al. (2016), this dataset has very good recall (i.e., producer's accuracy, which is the fraction of true plantations that have been detected over the total amount of true plantations) but has poor precision (i.e., user's accuracy, which is the fraction of true plantations among the detected plantations). RSPO dataset is available for three different years namely 2001, 2005, and 2009. This product provides a very detailed map with 19 classes for each of these years. This dataset has higher precision but poor recall. Even though these datasets are imperfect, they can serve as different sources of noisy labels that can be used for training machine learning models.

Although we have a few manually created plantation maps as sources of labels, the automated detection of land cover changes to/from plantations using remote sensing datasets is still a challenging task due to various reasons:

**High multi-modality**: There exists a wide variety of land cover types on the earth's surface. Different taxonomies have been defined to categorize locations on the earth's surface at different level of granularity. For example, Table 1 shows three different taxonomies. Similarly, more detailed or coarse taxonomies can be defined depending on the application. Ideally, we wish to use the most detailed taxonomy to categorize the locations to obtain the maximum information about land cover change. But in practice, learning classification models that can distinguish all these classes is difficult. There exists no high accuracy map that can provide sufficient high quality training samples for all these classes. In this work, we aim to simplify the problem by using a coarse taxonomy. Specifically, we aggregate all land cover types into three classes namely, Forests, Plantations and all the remaining land cover types are labeled as Other land cover class. Forest class has been kept separate from the group of rest of the classes because we are also interested in estimating how many of the plantations were established by removing forests. Now each of these three classes have subclasses within them which makes these classes highly multi-modal in nature. Hence, we need training samples from all these modes in order to achieve better classification accuracy.

**Table 1:** Correspondence between the aggregated classes defined in this paper, and high-level classes and land cover types in the RSPO dataset (see section 3.2.2).

**Aggregated**	**High-level class**	**RSPO land cover**	**Description**
Plantation	Oil palm	Oil palm plantation	Large industrial estates planted to oil palm
Plantation	Timber plantation	Timber plantation	Large industrial estates with timber or pulp species
Plantation	Agriculture	Rubber plantation	Large/medium sized industrial estates with rubber
Other	Agriculture	Coastal fish pond	Permanently flooded open areas
Other	Agriculture	Dry cultivated land	Herbaceous vegetation for row crops/pasture
Other	Agriculture	Mixed tree crops	Mosaic of cultivated and fallow land
Other	Agriculture	Rice fields	Rice paddy with seasonal or permanent inundation
Other	Built-up	Settlements	Villages, urban areas, industrial areas, open mining
Other	Mining	Mining	Open area with surface mining activities
Other	Bare soil	Upland grassland	Open vegetation dominated by grasses
Other	Bare soil	Upland shrub land	Open woody vegetation with forest and grassland
Other	Bare soil	Swamp grassland	Extensive herbaceous plants with shrubs/trees
Other	Bare soil	Swamp shrub land	Open woody vegetation on poorly drained soils
Other	Water body	Water bodies	Rivers, streams, and lakes
Other	Disturbed forest	Disturbed mangrove	Forest of mangrove species with clearing
Other	Disturbed forest	Disturbed swamp forest	Swamp forest with logging and clearings
Forest	Disturbed forest	Disturbed upland forest	Basal area reduced significantly due to logging
Forest	Undisturbed forest	Undisturbed upland forest	Natural forest, diverse species, and basal area
Forest	Undisturbed forest	Undisturbed swamp forest	Natural forest with inundation

*The last column provides a brief description of each land cover type*.

**Noisy ground truth**: In traditional classification settings, it is assumed that high quality ground truth labels are available for training the classification model. However, in this scenario high quality ground truth is not available. Instead, noisy labels from different sources are available. Hence, traditional classification techniques might have limited performance in this scenario.

**High dimensionality**: Most land cover classes have annual growth cycle and hence show a seasonal pattern. Classification cannot be done by using just a single time step as the separability between classes vary across the year. For example, a crop field just after harvest would look very similar to a barren land and hence would not be distinguishable. In order to achieve better classification performance, we need to incorporate both spectral properties at individual timesteps and the temporal pattern of growth of different land cover types.

**Spatial and temporal heterogeneity**: Due to atmospheric disturbances and natural variability in land cover types, classification models learned in a specific region for a specific time may not perform well when applied to other regions and time. Hence, there is a need to incorporate variations in feature space for better performance.

In this paper, we study the effectiveness of a machine learning framework to map plantation at annual scale. This framework learns a multi-class ensemble from noisy ground truth data obtained by manual labeling (i.e., TP and RSPO). The key contribution of this work is to study the effectiveness of several components in this framework by comparing the performance for a set of variants of this framework.

We compare the quality of annual plantation extents generated by the proposed framework with the existing datasets in the Kalimanthan region of Indonesia that were used for training our algorithms. Specifically, we have analyzed the portion of Kalimanthan that overlaps with MODIS tile h29v09. Through visual inspection of high resolution imagery and manually labeled set of points, we show that the proposed framework can overcome the imperfectness of the available products and thus has the potential to produce high-quality large-scale plantation maps with little manual effort.

## 2. Related Work

A wide variety of methods have been proposed that use remote sensing at different spatial and temporal scale for monitoring changes in land cover. However, a vast majority of these methods focus on detection deforestation activity only (Hansen et al., 2008; Hoscilo et al., 2011; Margono et al., 2012). The widely used global deforestation product (Hansen et al., 2013) does not differentiate between forest and plantations. Similarly, land cover product from NASA also does not model the plantation class separately.

Methods focused on detection conversions of land cover types to/from plantations have several issues that make them unsuitable for monitoring plantation related activities at large scale. Some methods involve extensive human involvement as they use visual interpretation in the detection process (Miettinen et al., 2012a,b; Ziegler et al., 2012). Few automatic machine learning based methods have also been proposed in the literature but they use very simple techniques such thresholding (Dong et al., 2012; Gutiérrez-Vélez and DeFries, 2013), nearest neighbor method (Li and Fox, 2012). Some sophisticated machine learning methods have only shown success in selected small-scale test dataset (Jia et al., 2017a,b). Due to these reasons current state of the art methods have limited applicability.

## 3. Study Region and Dataset

### 3.1. MODIS Data and Region of Study

**Figure 1:**
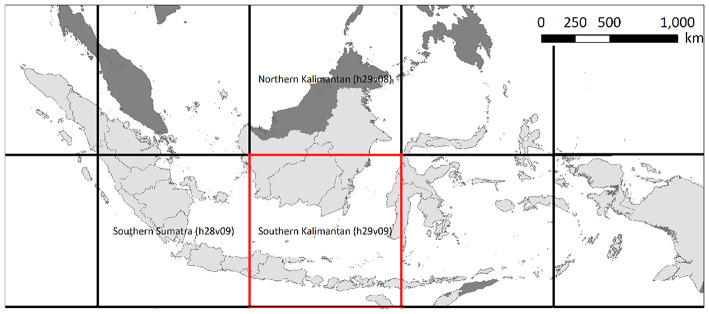
Our study region (marked in red color) in MODIS tile h29v09 (southern Kalimantan).

In this paper we utilize the MODIS MOD09A1 dataset which contains the seven-band reflectance values collected by MODIS instruments onboard NASA's Terra satellites. The remote sensing data in MODIS dataset are available at 500m resolution for every day. The daily images are then processed to generate 8-days composite images by selecting each location's reflectance value with least noise from the corresponding 8-days interval. We will validate the proposed approach on MODIS tile h29v09, as shown in Figure 1, which is a plantation-intensive region in southern Kalimanthan region of Indonesia (Abood et al., 2015; STA, 2018). This region contains 1,312,112 locations (i.e., MODIS pixels) at 500m spatial resolution, or equivalently 328,028 km^2^.

### 3.2. Ground-Truth Datasets

#### 3.2.1. Tree Plantation Dataset

Tree Plantation dataset (TP) is created by Transparent World and is available on Global Forest Watch. In this dataset, the plantation locations are labeled based on Landsat images circa 2013–2014 (Petersen et al., 2016), and each location is further categorized as industrial plantation, medium-sized plantation mosaic, small-sized plantation mosaic or very young plantations. This dataset contains in total 260,483 locations in our region of study. According to the visual inspection conducted by Petersen et al. (2016) and our comparison with high-resolution images from DigitalGlobe, the TP dataset covers most real plantation areas (high recall) but contains many mistakes (low precision).

#### 3.2.2. RSPO Dataset

RSPO dataset is provided by Roundtable on Sustainable Palm Oil (RSPO) Gunarso et al. (2013), and covers all the locations in the region of study. In this region, each location is categorized into one of 19 land cover types on 2000, 2005 and 2009 by RSPO dataset. Besides, it aggregates the 19 land cover types into 9 high-level classes, as described in Table 1. In a nutshell, RSPO dataset provides the information of plantation and other land cover types on 2000, 2005 and 2009 (see Table 2). Although the RSPO report (Gunarso et al., 2013) did not provide an accuracy assessment, our study on DigitalGlobe high-resolution images shows that RSPO dataset is accurate (high precision), but misses many real plantation areas (low recall).

**Table 2:** Count of MODIS pixels by land cover for the years 2000, 2005, and 2009 (columns 3–5), and the estimated area (10^3^ km^2^) of each land cover type for the years 2000, 2005, and 2009 (columns 6–8) for MODIS tile h29v09 (southern Kalimantan), reported by the RSPO dataset.

**Full name**	**Land cover**	**2000**	**2005**	**2009**	**A_**2000**_**	**A_**2005**_**	**A_**2009**_**
Coastal fish pond	CFP	5,120	5,159	6,324	1.28	1.29	1.58
Rubber plantation	CPL	18,398	19,813	19,741	4.60	4.95	4.94
Dry cultivated land	DCL	44,640	57,555	86,230	11.16	14.39	21.56
Disturbed upland forest	DIF	413,561	404,786	386,326	103.39	101.20	96.58
Disturbed mangrove	DIM	6,731	6,731	6,500	1.68	1.68	1.63
Disturbed swamp forest	DSF	81,790	83,001	66,836	20.45	20.75	16.71
Upland grassland	GRS	14,772	12,026	12,273	3.69	3.01	3.07
Mining	MIN	1,249	2,308	4,168	0.31	0.58	1.04
Mixed tree crops	MTC	6,944	7,657	7,995	1.74	1.91	2.00
Oil palm plantation	OPL	27,948	42,572	101,806	6.99	10.64	25.45
Rice fields	RCF	28,697	29,416	30,419	7.17	7.35	7.60
Upland shrub land	SCH	288,002	294,930	258,922	72.00	73.73	64.73
Settlements	SET	2,776	2,839	2,840	0.69	0.71	0.71
Swamp grassland	SGR	16,713	13,887	13,525	4.18	3.47	33.8
Swamp shrub land	SSH	98,669	103,509	108,240	24.67	25.88	27.06
Timber plantation	TPL	12,008	12,531	12,117	3.00	3.13	3.03
Undisturbed upland forest	UDF	136217	115656	97007	34.05	28.91	24.25
Undisturbed swamp forest	USF	88,069	77,928	71,035	22.02	19.48	17.76
Water bodies	WAB	19,808	19,808	19,808	4.95	4.95	4.95

## 4. Methods

In this section, we will first describe the proposed ensemble learning framework. We then introduce several baselines methods which are variants of our proposed method. By comparing against these baselines, we are able to demonstrate the effectiveness of each component in the proposed framework.

### 4.1. The Ensemble Learning Framework

The mapping of plantation is difficult from machine learning perspective since it requires the differentiation between plantation and multiple land cover types. If we directly merge all the non-plantation classes, such as evergreen forest, grassland, and cropland, as the negative class and conduct a binary classification between plantation and non-plantation, the heterogeneity within the negative class will greatly hamper the classification performance. There have been many existing works on multi-class classification (Angulo et al., 2003; Jia et al., 2019) and class heterogeneity (Pavlidis et al., 2001; Karpatne et al., 2014). However, these works cannot be directly adapted to our problem due to the skewness of different land cover types and the relationship among them. Besides, the complex feature space in remote sensing data poses a challenge for the learning process.

To solve these challenges, the proposed framework learns an ensemble model among multiple land cover classes. Specifically, we define three classes in the learning process: “plantation,” “forest,” and “other.” Specifically, the “plantation” class contains multiple types of plantations and “forest” class contains both the undisturbed forest and the forest with crossing roads but not yet logged. These three classes are obtained by aggregating the RSPO land cover types, as described in Table 1. Here we separately model the class of “forest” for two reasons. First, it is a well-known challenge to distinguish between plantation and forest, since oil palm trees can become as green as forest when they grow into mature phase. The other land cover types, such as urban area and cropland usually show lower level of greenness and therefore are easier to distinguish from plantation. Second, by identifying forest and plantations, we can better understand the conversion from tropical forests to plantations.

To learn the discriminative knowledge between each pair of classes, we propose to train three binary classifiers: “plantation” vs. “forest” (P-F), “forest” vs. “others” (P-O), and “others” vs. “plantation” (O-F). In this way each classifier focuses on exploiting the discriminative knowledge between a specific pair of classes. This learning strategy can greatly reduce the class heterogeneity and improve the learning performance. Since each binary classifier focuses on differentiating between a specific pair of classes, there are in total eight possible combinations of the outcomes from the three classifiers. Based on the separate prediction, we will assign the aggregated prediction result as the majority class label. For instance, if the classifiers of both P-F and O-P predict a test location as “plantation,” then we will label this test location as “plantation” regardless of the prediction of F-O classifier. We summarize the relationship between each individual prediction and the aggregated prediction in Table 3. In particular, when the three binary classifiers generate mutually different labels, as shown in the last two rows, we will label the test sample as “Unknown” (U). It is noteworthy that in our problem we are interested in detecting the coverage of plantation, which is marked by the first two rows.

**Table 3:** Aggregation of predictions from pair-wise classifiers (P, plantation; F, forest; O, other; U, unknown).

**P-F**	**F-O**	**O-P**	**Aggregation**
P	F	P	**P**
P	O	P	**P**
F	F	O	F
F	F	P	F
P	O	O	O
F	O	O	O
P	F	O	U
F	O	P	U

*We label a location as unknown (U) only when three individual pair-wise classifiers generate mutually different labels. We are interested in detecting plantations, which is marked by the first two rows (in bold)*.

To better extract useful discriminative knowledge from multi-temporal remote sensing data, we train a four-layer Deep Belief Networks (DBN) (Hinton, 2009) for each binary classifier (with 158, 64, 20 hidden variables, the last layer outputs the class label). We feed each DBN model with the concatenation of seven-band spectral features collected for 46 dates of a year. The model then outputs a class label for every pixel every year.

Another major challenge is that different land cover types can be highly skewed in real-world dataset. The training process is very likely to be dominated by the land cover types with large population, such as bare soil, if we adopt a uniform sampling strategy. To this end, we simultaneously sample equal amount of samples for each sub-classes within each aggregated class. Moreover, the training data are sampled from multiple years based on the RSPO dataset and the TP dataset.

Finally, we utilize a Hidden Markov Model (HMM) to post-process the classification outputs obtained from the ensemble model. The HMM model is able to capture common land cover transitions and fix a false classification label based on its previous labels. Consider a yearly sequence of {forest, forest, plantation, plantation, forest, plantation}, the “forest” at the fifth position is highly likely to be a classification error and should be fixed to “plantation” since plantations are rarely converted back to forests.

### 4.2. Comparison With Baseline Methods

To show the effectiveness of each component in our proposed framework, we compare to a set of baselines which cover different learning strategies (i.e., class aggregation, training data sampling, and learning model) and different post-processing methods.

#### 4.2.1. Different Learning Strategies

Here we compare with methods using different class aggregation, training data sampling and learning model selection.

- Binary: In this baseline, we train a binary classifier between plantation and non-plantation. Compared with the proposed three-class classification strategy, this baseline merges “forest” and “other” classes into non-plantation.
- Four-class: Here instead of using three aggregated classes, we define four classes: “plantation,” “forest,” “bare soil,” and “other.” The “bare soil” is defined based on the RSPO dataset (see Table 1). Then we will train 6 binary classifiers between each pair of classes. Similar with the proposed method, we aggregate the prediction result by majority voting.
- Uniform: In this baseline we uniformly sample from each aggregated class “plantation,” “forest,” and “other” rather than taking equal amount of samples from each sub-class.
- SVM: Instead of DBN, we implement our ensemble learning strategy using Support Vector Machine (SVM) with RBF kernel.

#### 4.2.2. Different Post-processing Strategies

In the proposed framework, we utilize the 19 land cover types defined in RSPO (see Table 1) as the latent classes in the HMM for post-processing. We wish to compare to the post-processing strategies using different settings for the HMM model. The HMM model is expected to model less complex transition patterns if we use less number of latent classes.

- NonP: This baseline is the same with the proposed learning method except that it does not involve the post-processing process.
- HMM9: Here we use HMM to conduct post-processing based on 9 higher-level aggregated classes provided in the RSPO dataset.
- HMM3: Here we use HMM to conduct post-processing based on the three aggregated classes—P, F, and O.

Here we introduce the involved metrics in measuring the performance. Since our proposed method generates yearly plantation map, we can measure the performance on each year. Specifically, we will measure the performance in terms of recall on each year from 2001 to 2009. The yearly recall is computed as the proportion of the “confident plantation locations” being successfully detected. A location is marked as “confident plantation location” if it is labeled as plantations by RSPO (available on 2000, 2005, and 2009) in both neighboring years from {2000, 2005, 2009}. For example, if a location is labeled as plantations by RSPO in both 2000 and 2005, it is a “confident plantation location” for every year from 2000 to 2005.

Since the RSPO dataset has low recall, we cannot use the RSPO dataset to estimate the precision in each year. Instead, we measure the overall precision using the Tree Plantation (TP) dataset (in 2014) because TP has high recall and thus any locations that are not labeled by TP are unlikely to be plantations. We also measure the overall recall using the RSPO dataset (on 2009). The overall precision and recall are measured using all the detected plantation locations through 2001 to 2014. More formally, the overall precision measures the fraction of plantations that are labeled both by our method and by the TP dataset over all the detected plantations by our method. The overall recall measures the fraction of plantations that are labeled both by our method and by the RSPO dataset in 2009 over all the plantations labeled by the RSPO dataset in 2009.

## 5. Experiments and Results

### 5.1. Plantation Maps

**Figure 2:**
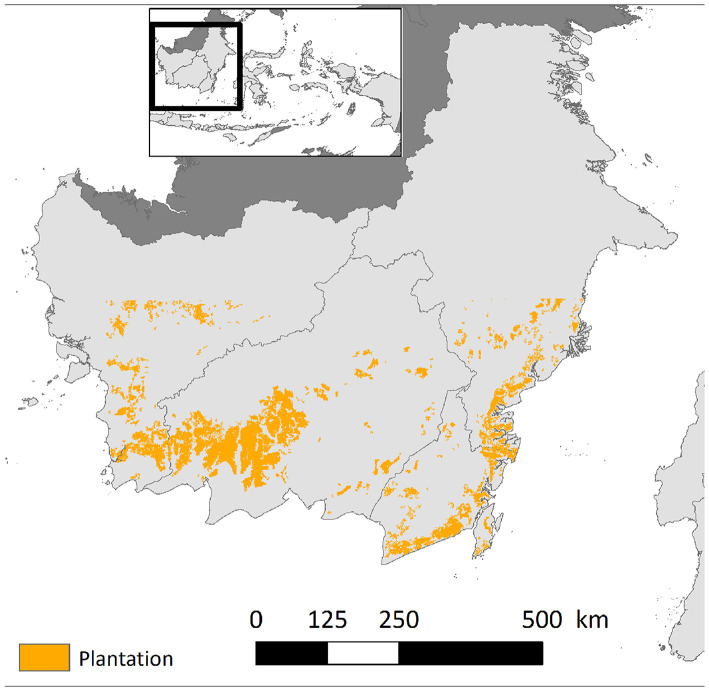
The generated plantation maps in 2014. The plantation locations are marked in yellow color.

**Figure 3:**
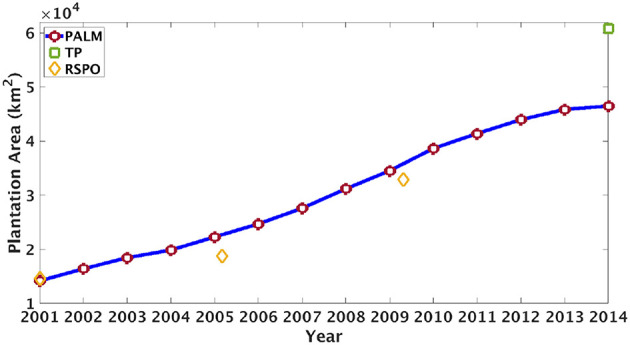
The annual plantation area (km^2^) detected by PALM in our study region (southern Kalimantan) from 2001 to 2014. The area of plantations labeled by TP is shown in 2014 and the area of plantations detected by RSPO is shown in 2000, 2005, and 2009.

Based the proposed method we can generate yearly plantation maps. For instance, we show our generated plantation maps in 2014 in Figure 2 and the growing area of plantations in Figure 3. According to our detection results, the plantation area in this region has an average annual increase of around 10%. According to Figure 3 our method detects more plantations than the RSPO dataset but much less plantations than the TP dataset. In section 5.5 we will show several examples to study the difference between our detection and existing products.

### 5.2. Comparison of Learning Strategy

From the results shown in Table 4, we can validate the effectiveness of each component in the proposed method. First, we can observe that the binary classification Binary leads to unsatisfactory performance due to the strong heterogeneity within the non-plantation class. Besides, Four-class leads to less precision than the proposed method, since we have more complex combinations based on the predictions from the 6 classifiers, and the ensemble learning result can be less confident. Moreover, the performance of Uniform is not as good as our approach since the training is dominated by the land cover types with large population (e.g., forests, croplands), and ignores the small classes (e.g., bare soil, mining) that are similar to plantation. In this way the trained classifier is highly likely to misclassify these small classes as plantation, and consequently leads to low precision. Furthermore, we can observe that the proposed method outperforms SVM by a considerable margin due to the effectiveness of DBN in extracting discriminative patterns from complex feature space.

**Table 4:** Comparison to different learning strategies (see section 4.2), using the yearly recall from 2001 to 2009, overall precision and overall recall.

**Method**	**2001**	**2002**	**2003**	**2004**	**2005**	**2006**	**2007**	**2008**	**2009**	**Pre**	**Rec**
Binary	0.7334	0.7822	0.8041	0.8130	0.8210	0.7665	0.7809	0.7975	0.8234	0.8351	0.8431
Four-class	0.8052	0.8315	0.8571	0.8691	0.8801	0.8253	0.8411	0.8581	0.8732	0.8282	0.8679
Uniform	0.7516	0.8063	0.8275	0.8404	0.8489	0.7992	0.8142	0.8318	0.8474	0.7830	0.8619
SVM	0.5436	0.6537	0.7196	0.7592	0.7800	0.7364	0.7490	0.7562	0.7601	0.7365	0.6428
Proposed	0.7586	0.8164	0.8420	0.8531	0.8674	0.8099	0.8229	0.8374	0.8577	0.8463	0.8677

*The yearly recall measures the fraction of plantations that are detected within “confident plantation locations” (by RSPO) over the total number of “confident plantation locations” in each year. The overall precision measures the fraction of detected plantations that are also labeled by the TP dataset in 2014 over all the detected plantations. The overall recall measures the fraction of detected plantations that are also labeled by the RSPO dataset over all the plantations labeled by the RSPO dataset in 2009*.

### 5.3. Comparison of Post-processing Steps

We show the performance of the proposed method and the baselines with different post-processing strategies in Table 5. First, the comparison between NonP and other methods demonstrates the effectiveness of post-processing. Besides, the HMM using 19 land cover classes outperforms the HMM model with nine latent classes or three latent classes. This is because the 19 classes can better define the latent state space in HMM and more accurately model the transition process. Using less latent classes is equivalent to merging multiple different transitions to be a single transition. The resulted heterogeneity in the merged transition patterns is likely to degrade the performance.

**Table 5:** Comparison to different post-processing strategy (see section 4.2), using the yearly recall from 2001 to 2009, overall precision and overall recall.

**Method**	**2001**	**2002**	**2003**	**2004**	**2005**	**2006**	**2007**	**2008**	**2009**	**Pre**	**Rec**
NonP	0.7670	0.7573	0.7856	0.7709	0.8284	0.7403	0.7431	0.7609	0.8153	0.7501	0.8331
HMM9	0.7208	0.7894	0.8181	0.8393	0.8600	0.8056	0.8221	0.8406	0.8658	0.8253	0.8801
HMM3	0.8766	0.8766	0.8766	0.8800	0.8859	0.8308	0.8385	0.8476	0.8616	0.8269	0.8606
Proposed	0.7586	0.8164	0.8420	0.8531	0.8674	0.8099	0.8229	0.8374	0.8577	0.8463	0.8677

*The yearly recall measures the fraction of plantations that are detected within “confident plantation locations” (by RSPO) over the total number of “confident plantation locations” in each year. The overall precision measures the fraction of detected plantations that are also labeled by the TP dataset in 2014 over all the detected plantations. The overall recall measures the fraction of detected plantations that are also labeled by the RSPO dataset over all the plantations labeled by the RSPO dataset in 2009*.

### 5.4. Performance Using Different Amounts of Training Data

We also examine the relationship between classification performance and amount of training data. Specifically, we will test the performance in 2001, 2005, 2009, and 2013 using different amount of training data. The performance is measured using a separate testing set of 3,000 confident plantation samples and 3,000 non-plantation samples (i.e., the union of “forest” and “other”).

**Figure 4:**
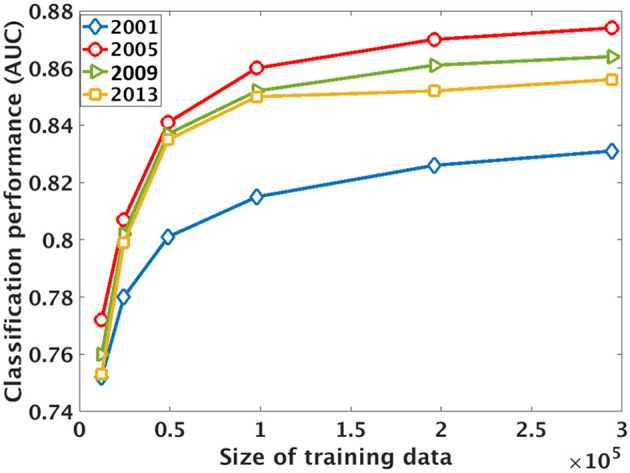
The classification performance [area under ROC curve (AUC)] with respect to the size of the training dataset. X-axis shows the total number of samples for the combined plantation, forest, and other classes. The performance is measured on a separate testing set with 6,000 samples. Each curve shows the performance in a specific year.

It can be seen that there is a strong positive and non-linear relationship between the quantity of training data and model performance (Figure 4). The performance increases rapidly when the data size is small, but increases slowly after the data size is larger than 10,000.

We conclude that plantation mapping is challenging because effective training of a classification model for detecting plantations in a new region requires sufficient manually labeled samples. Only by learning from sufficient samples can the model extract discriminative patterns that distinguish plantations with all the other land covers.

### 5.5. Visual Verification Using High Resolution Data

As mentioned earlier, Tree Plantation has high recall but low precision, while RSPO has high precision but low recall. Here we wish to show our generated plantation maps can achieve a better balance than these two ground-truth datasets. We verify this by using high-resolution DigitalGlobe data. Specifically, we study three different cases.

**Figure 5:**
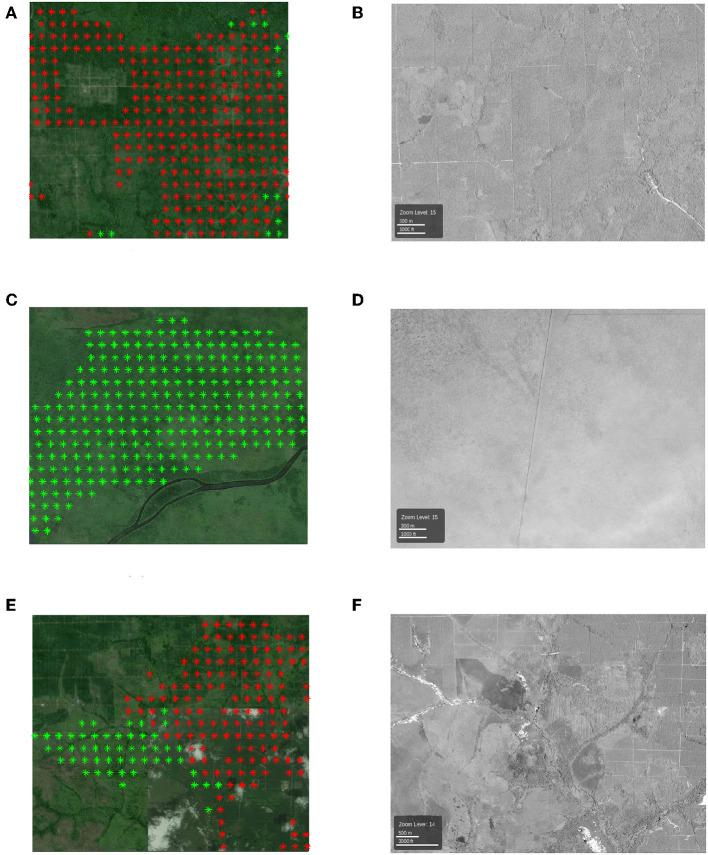
**(A,C,E)** The examples of locations that are labeled as plantations by TP but not by RSPO. The red color denotes the locations that labeled by the proposed method and TP, and the green color denotes the locations that are labeled only by TP. Each colored point is the center of a 500 m-by-500 m pixel. **(B,D,F)** High-resolution DigitalGlobe images (north at the top) in the same area with **(A,C,E)**.

*1. The locations that are labeled as plantations by TP but not by RSPO:* To analyze this scenario, we show three examples in Figure 5. Here the red color represents the locations in that are labeled as plantations by the proposed method and TP but not by RSPO, and the green color represents the locations that are labeled only by TP. We show the high-resolution images corresponding to Figures 5A,C,E using DigitalGlobe in Figures 5B,D,F, respectively.

According to the high-resolution image, the red colored region in Figure 5A is a real plantation area, but is missing from the RSPO dataset. As for the green colored region in Figure 5C, which is included by Tree Plantation dataset but not detected by our method, we can clearly see from the high-resolution image that it is not real plantation. In Figure 5E we show an area with locations in both red and green colors. From the high-resolution image in Figure 5F, we can observe that the proposed method can well detect the boundary between real plantation and non-plantation area.

With these examples in R1 and R2, we demonstrate that our proposed method can detect the real plantation locations that are missing from the RSPO dataset while also avoiding the locations that are mistakenly detected by Tree Plantation dataset.

**Figure 6:**
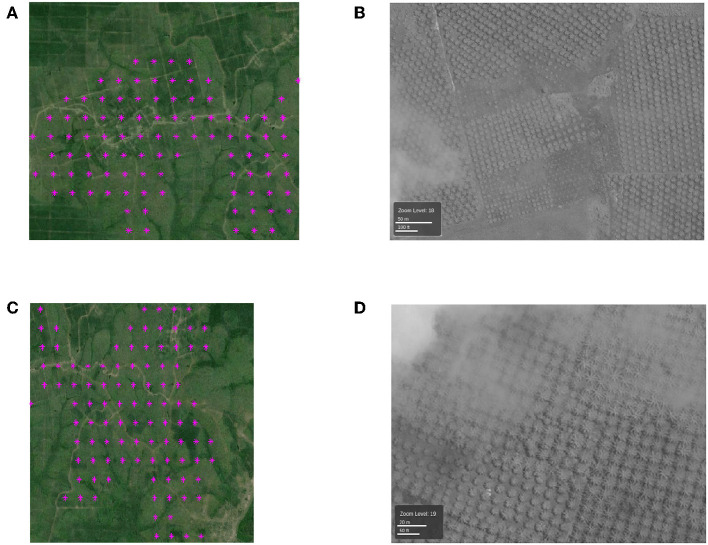
**(A,C)** The examples of locations that are labeled as plantations by the proposed method but not by TP (in magenta). Each colored point is the center of a 500 m-by-500 m pixel. **(B,D)** High-resolution DigitalGlobe images (north at the top) in the same area with **(A,C)**.

*2. The locations that are labeled as plantations by the proposed method but not by TP:* Now we take several examples for the case that are detected by our approach but missed from Tree Plantation dataset, as shown in Figures 6A,C. By using the corresponding high-resolution images (Figures 6B,D), we can clearly see that they are real plantation. In this way, we demonstrate that our method has a potential to detect true plantations that are not detected by the Tree Plantation dataset. Hence, our method can achieve high precision, which is even higher than the estimated precision using Tree Plantation dataset (0.8463).

**Figure 7:**
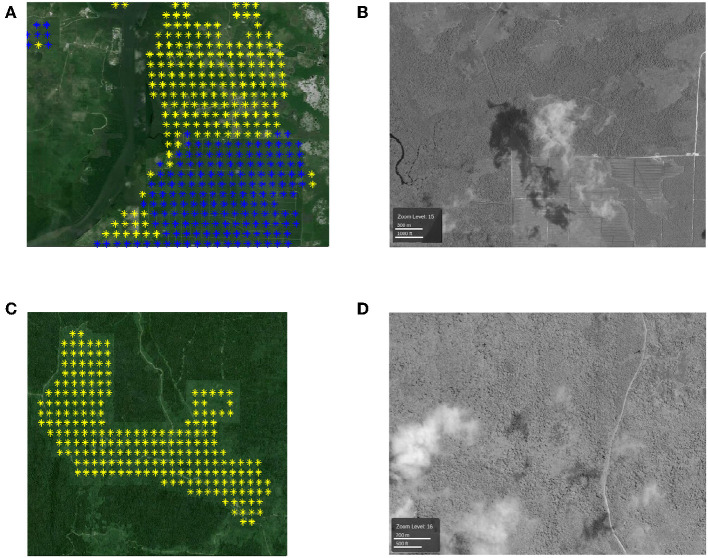
**(A,C)** Examples of locations that are labeled as plantations by RSPO but not by the proposed method (in yellow). The blue color denotes the locations that are detected by both RSPO and the proposed method. Each colored point is the center of a 500 m-by-500 m pixel. **(B,D)** High-resolution DigitalGlobe images (north at the top) in the same area with **(A,C)**.

*3. The locations that are labeled as plantations by RSPO but not by the proposed method:* We show several large example patches in Figures 7A,C and corresponding high-resolution images in Figures 7B,D, respectively. We show the locations that are labeled as plantations by RSPO but not by the proposed method in yellow color. According to our observation, these locations are usually adjacent to the locations that are included by both the RSPO dataset and our approach (in blue).

According to the corresponding high-resolution images, the locations in yellow color are not real plantation. This shows that some locations are mistakenly labeled as plantations by the RSPO dataset but are labeled correctly by our method. Hence, the actual recall of our proposed method is higher than the estimated recall value using the RSPO dataset (0.8677).

A fair and thorough validation of our generated plantation maps requires sufficient ground-truth plantation samples. While the visual validation of generated maps is beyond the scope of this paper, we used a sampling-based approach for a more detailed examination of locations discussed in the above three scenarios and measured the accuracy of the proposed method and existing plantation products. The results were discussed in our previous report (Jia et al., 2016).

## 6. Conclusion

In this paper, we study several key components in a machine learning framework for automatically creating plantation maps. These components includes class aggregation, data sampling, learning model selection, and post-processing. The evaluation of multiple baselines derived from this framework confirms the effectiveness of each component. The visual verification of the proposed framework on a large region in Indonesia (MODIS tile h29v09) shows that the proposed method can generate high-quality annual plantation maps and our detection achieves a better balance of precision and recall than those datasets that were used for training our proposed framework.

The methods we presented here can be used to create plantation mapping products. In future, we will make use of the generated plantation maps to understand how plantation conversion impacts the environment and better monitor the policy compliance.

For example, the analysis using a combination of generated plantation maps and auxiliary datasets, such as the Plantation Concessions Dataset^1^ has an implication on potential illegal plantation areas. We also plan to leverage the fire product (Mithal et al., 2018) to detect uncontrolled fires from plantation conversion and study their impact to deforestation. In addition, we will analyze the correlations between plantation dynamics and the carbon emission^2^ to study how cultivating plantations leads to large amount of carbon emissions.

Our proposed method also remains limited in terms of validation and imagery inputs, which need to be addressed in future work. The first limitation lies in that the reference data used in the validation process (e.g., TP, RSPO, and DigitalGlobe) are mostly created through manual inspection. However, some plantations may not be easily identified visually due to their advanced age and associated high tree cover. Second, our analysis was also limited by the resolution of the MODIS data. While the high resolution of Landsat data (30 m) and Sentinel data (10 m) offer potential to map plantations more accurately, the low temporal frequency of Landsat (16 days) and Sentinel (5/10 days) makes it hard to find images with little noise (e.g., clouds). A joint multi-scale learning framework has potential to better delineate the boundary of target classes with a higher spatial resolution while also taking advantage of rich temporal knowledge from more frequently collected satellite data.

## Data Availability Statement

The datasets for this manuscript are not publicly available because the RSPO dataset is currently private and is still being updated. Requests to access the datasets should be directed to jiaxx221@umn.edu.

## Author Contributions

XJ was the primary author who conducted most of the experiments and analysis. AK conducted some parts of the experiments. KC, JG, and PW were our domain experts who greatly helped in analyzing the results and also helped with the text. VK was the one who managed the entire team and also helped to review the work.

### Conflict of Interest

The authors declare that the research was conducted in the absence of any commercial or financial relationships that could be construed as a potential conflict of interest.

## Footnotes

^1^http://data.globalforestwatch.org/datasets/oil-palm-concessions?geometry=88.921%2C-9.085%2C163.232%2C6.245

^2^http://data.globalforestwatch.org/datasets/e129158a68434ca49d96a40d0e3109b1?geometry=94.614%2C-3.446%2C131.77%2C4.239
